# Bcl-2-Ome – a database and interactive web service for dissecting the Bcl-2 interactome

**DOI:** 10.1038/cdd.2016.129

**Published:** 2016-11-11

**Authors:** Annika Hantusch, Thomas Brunner, Tancred Frickey, Markus Rehm

**Affiliations:** 1 Department of Biology, Applied Bioinformatics, University of Konstanz, Konstanz 78457, Germany; 2 Department of Biology, Biochemical Pharmacology, University of Konstanz, Konstanz 78457, Germany; 3 Konstanz Research School Chemical Biology, University of Konstanz, Konstanz 78457, Germany; 4 Department of Physiology and Medical Physics, Royal College of Surgeons in Ireland, Dublin 2, Ireland; 5 Institute of Cell Biology and Immunology, University of Stuttgart, Stuttgart 70569, Germany; 6 Stuttgart Research Center Systems Biology, University of Stuttgart, Stuttgart 70569, Germany

*Dear Editor*,

The interactome of the Bcl-2 protein family regulates mitochondrial outer membrane permeabilisation and apoptotic cell death and, following decades of research, now takes center stage in the development of targeted anti-cancer therapeutics.^1, 2^ Bcl-2 family members physiologically interact in aqueous and lipid membrane environments and also at interfaces between both phases. Consequently, a broad range of methodological approaches is employed to study the Bcl-2 family interactome, with differences in experimental conditions and reaction environments strongly affecting interaction kinetics and steady states. Since detailed insight into the Bcl-2 family interactome is crucial not only for understanding signal transduction, but also for the further development of targeted therapeutics and the context-dependent identification of optimal interventions, tools are required that assist in navigating through the large amount of available, complex and frequently conflicting data. We address this need by offering a comprehensive database and interactive web service (Bcl-2-Ome) that provides structured, annotated and expert-curated data on the Bcl-2 interactome, a framework for prospective community-assisted data integration and database expansion, and a user-friendly interface for searches, data filtering and interactome visualization. All database entries are linked back to primary source literature and include detailed information on experimental conditions and reagents used for data generation. Bcl-2-Ome therefore allows the exploration of the Bcl-2 family interactome at an unprecedented level of convenience and detail. Bcl-2-Ome is implemented as a Java Web Start application and is freely accessible at http://for2036.uni-konstanz.de/Bcl2Ome/. All contents are available for download as.xml file, and the web service also links to less specific but global interactome resources (STRING, IntAct) and the BCL2DB repository for sequence and structural information, ensuring that Bcl-2-Ome is offered within the context of existing repositories.

By now, 353 quantitative and qualitative experimental outcomes are integrated in Bcl-2-Ome. Three hundred and six entries describe the outcome of interaction studies and 47 entries describe effector (Bax, Bak) activation, such as the transient interactions with BH3-only proteins. Bcl-2-Ome can be accessed and interrogated through a user interface, which provides comprehensive data exploration possibilities. Search and filter criteria can be defined, the Bcl-2 interactome is graphically illustrated accordingly and provides access to detailed underlying original data (Supplementary Figure 1). The web service provides step-by-step instructions for representative use cases online. Since Bcl-2-Ome enables the convenient comparison of findings from multiple studies, information on reproducibly reported interactions can easily be sourced. Likewise, poorly defined or conflicting data on interactions within the Bcl-2 family can be evaluated within the context of the different assays, reactants, reaction conditions and cellular models used in the original research studies. Through a data submission interface, Bcl-2-Ome allows community-assisted expansion and regular updating. Submissions are reviewed for completeness, and curated as needed before inclusion. Taken together, we therefore believe that Bcl-2-Ome and its functionalities will be useful to a wide community of cell death researchers that may approach the field from a background of cell biology, biochemistry, biophysics or systems biology.
