# Supplementary material for: Bcl-2-Ome – a database and interactive web service for dissecting the Bcl-2 interactome
Source: Cell Death Differ. 2016 Nov 11;24(1):192. doi: 10.1038/cdd.2016.129 (PMC5260498; doi:10.1038/cdd.2016.129)
Supplement: Supplementary Figures [file cdd2016129x1.docx]

**Supplemental Figure Legend**

**Supplemental Fig. 1. Graphical User Interface of the Bcl-2-Ome web service.** Bcl-2 family members are displayed as a graph on the left. Connecting lines (edges) represent individual experimental outcomes from the peer reviewed literature. Interactions and effector activation are denoted by undirected and directed edges, respectively. The example shows the interactions for the Bcl-2 family member Bcl-x_L_. Edges can be selected via mouse clicks, resulting in the corresponding experimental details being displayed in the lower right corner of the user interface. The information depicted therein describes the relation of the two proteins as identified in the respective experiment, the type of experimental approach taken to obtain the data, a Boolean value specifying whether an interaction/effector activation was observed in this experiment and the associated literature reference. A short description of the experimental outcome adds further details to each result. Depending on the experimental approach and the data availability, additional information are given: a quantitative value such as the K_D_ or IC_50_ value, information on the cell line, detergents, antibodies and instrumentation used. Finally, details on whether and which recombinant or endogenous proteins were used are given, including sequence information and origin of the sequences. The top right panel provides a set of filters that allow users to focus on subsets of the data, e.g. limit the display to only quantitative or qualitative results, or interactions between specific proteins and to include or exclude results obtained by specific methods. A color code assists in differentiating between experimental approaches. Numbers in brackets denote the number of entries present in the database for the respective experimental approaches. The lower part of the right panel contains additional tabs to select experiments by reference or to retrieve Bcl-2 protein sequences of human or mouse origin as defined by UniProt (http://www.uniprot.org). Detailed step-by-step instructions on how to operate the webservice are provided online.
