# Supplementary figures and images for: Bcl-2-Ome – a database and interactive web service for dissecting the Bcl-2 interactome
Source: Cell Death Differ. 2016 Nov 11;24(1):192. doi: 10.1038/cdd.2016.129 (PMC5260498; doi:10.1038/cdd.2016.129)

**Supplemental** **Fig.1**


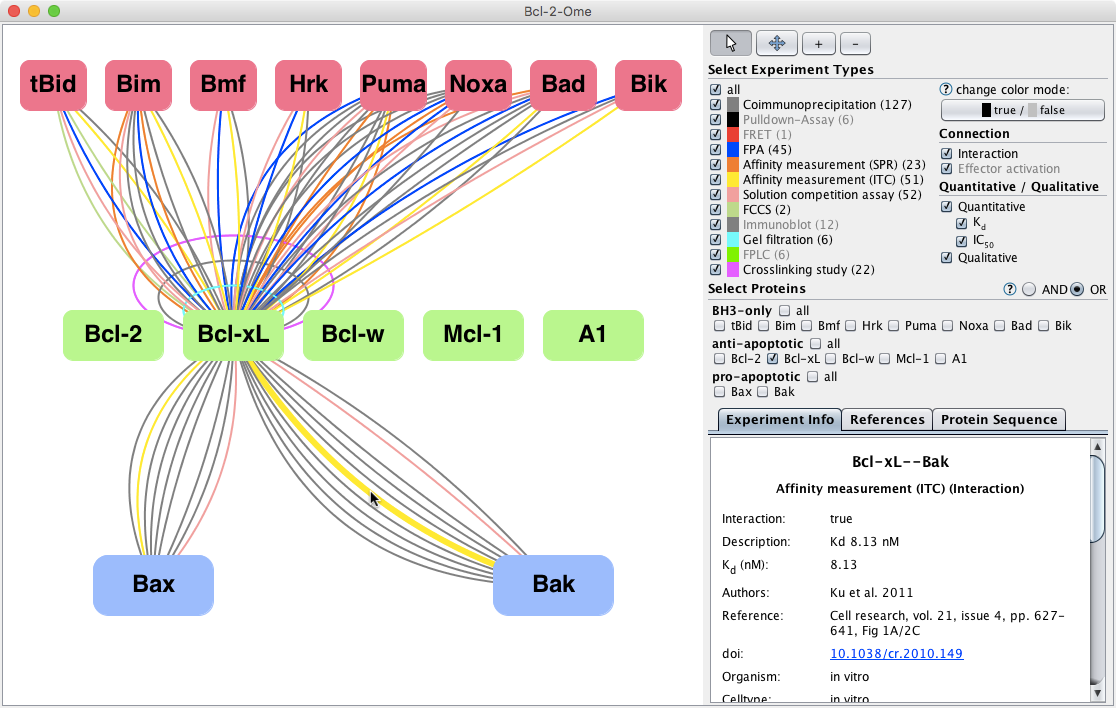

Supplement: Supplementary Figure 1 [file cdd2016129x2.docx]
